# The Relationship Between Renal Stones and Primary Aldosteronism

**DOI:** 10.3389/fendo.2022.828839

**Published:** 2022-02-09

**Authors:** Chun-Kai Chang, Chin-Chen Chang, Vin-Cent Wu, Jiun-Hung Geng, Hsiang-Ying Lee

**Affiliations:** ^1^ Department of Urology, Kaohsiung Medical University Hospital, Kaohsiung, Taiwan; ^2^ Department of Medical Imaging, National Taiwan University Hospital and National Taiwan University College of Medicine, Taipei, Taiwan; ^3^ Department and Graduate Institute of Forensic Medicine, National Taiwan University College of Medicine, Taipei, Taiwan; ^4^ Section of Nephrology, Department of Internal Medicine, National Taiwan University Hospital, Taipei, Taiwan; ^5^ Department of Urology, Kaohsiung Municipal Siaogang Hospital, Kaohsiung, Taiwan; ^6^ Department of Urology, School of Medicine, College of Medicine, Kaohsiung Medical University, Kaohsiung, Taiwan; ^7^ Graduate Institute of Clinical Medicine, College of Medicine, Kaohsiung Medical University, Kaohsiung, Taiwan

**Keywords:** kidney stone disease, nephrolithiasis, stone size, primary aldosteronism, essential hypertension

## Abstract

**Introduction:**

The association between primary aldosteronism (PA) and nephrolithiasis is still unclear. The hypercalciuria and hypocitraturia of PA patients might be the reason leading to recurrent calcium nephrolithiasis. This study aimed to evaluate the relationship between PA and renal stones, including stone size and density.

**Materials and Methods:**

From February 2010 to March 2021, we retrospectively collected 610 patients who presented to our medical center with hypertension history, and all these patients, suspicious of PA, had PA data survey. In total, 147 patients had kidney stone and were divided into 44 patients with essential hypertension as group 1 and 103 patients with PA as group 2. Pearson χ^2^ test and independent Student’s t-test were performed to examine the differences among variables.

**Results:**

The mean age was 54.4 ± 12.0 years in group 1 and 53.0 ± 11.1 years in group 2. The incidence rate of renal stones in the PA group was around 24%. No significant differences between the two groups were found for gender, systolic/diastolic blood pressure, duration of hypertension, diabetes mellitus history, and laterality of kidney stone; however, mean stone size was 4.0 ± 3.3 mm in group 1 and 6.5 ± 7.2 mm in group 2, with a significantly larger renal stone size noted in the PA group than that in the essential hypertension group (p = 0.004). Hounsfield unit (HU) density was higher in the PA group *vis-à-vis* the essential hypertension cohort, although this did not reach a significant difference (p = 0.204).

**Conclusions:**

Our study revealed that PA patients had a higher incidence rate of renal stones compared to that of the general population. Besides, the PA-related renal stones also presented as larger and harder than those of the essential hypertension group. Further investigation concerning the association between PA and renal stones is warranted.

## Introduction

In the past year, clinicians appear unfamiliar with severe hypertension or refractory hypertension recognized as essential hypertension that has only been controlled with several kinds of medication but somewhat ineffectively (1). In 1955, Dr. Conn firstly described the new entity called primary aldosteronism (PA) caused by overproduction of aldosterone; after that, physicians became aware of this problem (2). There are many subtypes of PA, the most frequent causes including bilateral adrenal hyperplasia (BAH), also known as idiopathic hyperaldosteronism (IHA), which accounts for 60%–70%, and aldosterone-producing adenoma (APA), which accounts for 30%–40% (3–5).

APA patients are younger (<50 years of age) than those with BAH. Besides, APA patients also have higher aldosterone secretion rates, resulting in higher plasma and urinary levels of aldosterone, leading to more severe hypertension and profound hypokalemia (4). The classic signs of PA are hypertension and hypokalemia, but more recently, more non-hypokalemic patients are being noticed (5, 6). PA also has some negative effects on cardiovascular (7, 8) and renal systems (9, 10), increasing the incidence of metabolic syndrome (11–13), and osteoporosis (14). In addition, several studies have illustrated the deleterious impact of PA on physical and mental quality of life (15).

Kidney stone disease (KSD), also known as nephrolithiasis, is a general urological issue, causing high cost and clinical burden to health care systems. In the United States, medical expenditures for KSD were over $2.1 billion in 2000 (16). A kidney stone starts small but can grow larger in size, even filling the inner hollow structure of the kidney although not causing any problem. Sometimes, the stone passes down the ureter, and if the stone gets stuck in the urinary tract, it could induce several symptoms such as sharp, cramping pain in the flanks, nausea, vomiting, and gross hematuria. The obstruction can further result in hydronephrosis, sepsis, and even acute renal failure; moreover, KSD is a risk factor for chronic kidney disease, cardiovascular disease, and osteoporosis (17–20). Preventing the incidence of KSD is an incrementally more significant issue, and stone formation has been proven as related to environmental and genetic factors such as climate, diet, fluid intake, smoking, caffeine, age, gender, body mass index (BMI), and type 2 diabetes mellitus (DM) (21–26).

According to the Urological Association of Asia clinical guideline for urinary stone disease, hypertension is not recognized as a risk factor of urolithiasis (27). On the contrary, PA might play an important role in renal stone. Some potential reasons may explain the association between PA and nephrolithiasis. Calcium metabolism controlled by some hormone may have a significant role in the connection of nephrolithiasis. Kabadi (28) mentioned the first case report about renal calculi as a major manifestation of PA in 1995. Increased urinary excretion of calcium might be the reason. Shey et al. (29) discussed a case report about recurrent calcium nephrolithiasis associated with PA and also disclosed the pathophysiology of hypercalciuria and hypocitraturia in hyperaldosteronism, which might be the cause of recurrent calcium nephrolithiasis. Although a definite association between PA and nephrolithiasis has not yet been clarified, the aim of this study is to elaborate the relationship between PA and nephrolithiasis in kidney stone size.

## Materials and Methods

From February 2010 to March 2021, 610 patients who had hypertension history in the National Taiwan University Hospital were retrospectively identified with all the patients, suspicious of PA, having undergone PA survey including plasma aldosterone concentration (PAC), plasma renin activity (PRA), aldosterone-to-renin ratio (ARR), and abdomen computed tomography (CT). Confirmatory testing was also done in each patient for PA diagnosis. Basic information such as age, gender, BMI, systolic/diastolic blood pressure (SBP/DBP), duration of hypertension, and DM was collected, as was laboratory data including potassium level and estimated glomerular filtration rate (eGFR). Additionally, PA patients were also divided into BAH and APA cohorts. Finally, 147 patients (44 patients with essential hypertension and 103 patients with PA) with renal stone were included in this study, with approval secured from our institutional review board [KMUHIRB-E(II)-20180184 and NTUH-REC No. 200611031R].

The length (maximum diameter), number, and location of renal stones were determined using CT. The mean Hounsfield unit (HU) value of the stones was calculated by averaging CT attenuation values at the center and outermost edge of the stone. In patients with multiple stones, stone diameter was considered as the sum of each stone, whereas the mean HU value was the average of the mean HU value of each stone. All measurements were calculated by radiologists.

Categorial variables are presented as percentages and continuous variables as mean ± standard deviation. Pearson χ2 test was performed to examine the differences among categorical variables, while independent Student’s t-test examined the differences among continuous variables. SPSS 20.0 (IBM Corp., Armonk, NY, USA) was used to perform the analyses, with a p value <0.05 regarded as statistically significant.

## Results

One hundred forty-seven patients with renal stone, including 44 patients (23.3%) in the essential hypertension group (group 1) and 103 patients (24.5%) in the PA group (group 2), were enrolled in the study as shown in **Table 1**, with demographic characteristics shown in **Table 2**. The mean age was 54.4 ± 12.0 years in group 1 and 53.0 ± 11.1 years in group 2, while 31 (71%) persons in group 1 and 61 (59%) persons in group 2 were males. Patients with DM were 5 (11%) persons in group 1 and 24 (23%) persons in group 2, while BMI levels (kg/m^2^) were 27.3 ± 6.5 in group 1 and 25.5 ± 3.7 in group 2, with a significantly lower BMI level in the PA group than in the essential hypertension group (p = 0.042). Mean SBP, DBP, and duration of hypertension were 147.2 ± 29.5 mmHg, 90.4 ± 19.9 mmHg, and 5.9 ± 5.7 years in group 1 and 154.7 ± 17.7 mmHg, 93.2 ± 11.9 mmHg, and 6.9 ± 6.6 years in group 2, respectively.

**Table 1:** The numbers of subjects with kidney stone disease divided by essential hypertension and primary aldosteronism.

Characteristics	Essential hypertension N = 189	Primary aldosteronism N = 421	Total N = 610
Stone, n (%)	44 (23.3)	103 (24.5)	147 (24.1%)

**Table 2:** Demographic and clinical characteristics of the subjects with kidney stone disease divided by essential hypertension and primary aldosteronism.

Characteristics	Subjects with essential hypertension N = 44	Subjects with primary aldosteronism N = 103	p value
Age (years)	54.4 ± 12.0	53.0 ± 11.1	0.502
Male, n (%)	31 (71)	61 (59)	0.264
BMI (kg/m^2^)	27.3 ± 6.5	25.5 ± 3.7	**0.042**
Systolic BP (mmHg)	147.2 ± 29.5	154.7 ± 17.7	0.058
Diastolic BP (mmHg)	90.4 ± 19.9	93.2 ± 11.9	0.391
Duration of hypertension (year)	5.9 ± 5.7	6.9 ± 6.6	0.353
Diabetes mellitus, n (%)	5 (11)	24 (23)	0.116
PAC (ng/dl)	36.9 ± 29.0	54.4 ± 39.6	**0.009**
PRA (ng/ml/h)	2.1 ± 2.1	0.9 ± 4.0	0.062
ARR (ng/dl per ng/ml/h)	222.8 ± 622.4	527.1 ± 1533.4	0.206
PAClog	1.5 ± 0.3	1.7 ± 0.3	**0.001**
PRAlog	-0.1 ± 0.8	-0.6 ± 0.6	**0.001**
ARRlog	1.6 ± 0.8	2.2 ± 0.6	**0.001**
K	4.1 ± 0.6	3.5 ± 0.6	**<0.001**
eGFR	85.0 ± 23.8	87.8 ± 30.7	0.552
Stone side (Bilateral, Left, Right)	10 (22), 17 (39), 17 (39)	34 (33), 42 (40), 27 (27)	0.374
Stone size (mm)	4.0 ± 3.3	6.5 ± 7.2	**0.004**
Stone (HU)	223.8 ± 159.6	267.7 ± 203.7	**0.204**

BMI, body mass index; BP, blood pressure; PAC, plasma aldosterone concentration; PRA, plasma renin activity; ARR, aldosterone-to-renin ratio; K, potassium; eGFR, estimated glomerular filtration rate; HU, Hounsfield unit. A p value < 0.05 is regarded as statistically significant (shown in bold).

The mean PAC (ng/dl), PRA (ng/ml/h), and ARR (ng/dl per ng/ml/h) levels were 36.9 ± 29.0, 2.1 ± 2.1, and 222.8 ± 622.4 in group 1 and 54.4 ± 39.6, 0.9 ± 4.0, and 527.1 ± 1,533.4 in group 2, respectively, with a significantly lower PAC level in the essential hypertension group than that in the PA group (P = 0.009). Owing to the small number of patients without distributed variables, log transformation for PAC, PRA, and ARR levels was used. Logs of PAC, PRA, and ARR levels all showed a significant difference with p = 0.001. The mean potassium level was 4.1 ± 0.6 in group 1 and 3.5 ± 0.6 in group 2, and lower potassium level was noted in PA group (p < 0.001). The mean stone size was 4.0 ± 3.3 mm in group 1 and 6.5 ± 7.2 mm in group 2, while a significantly larger renal stone size was noted in the PA group than that in the essential hypertension group (p = 0.004). The mean HU of the stone was 223.8 ± 159.6 in group 1 and 267.7 ± 203.7 in group 2. Although a significant difference was not revealed, the tendency that the PA group scored higher in stone size than the essential hypertension group was noted.

As listed in **Table 3**, the PA group was separated into BAH group (group 3) and APA group (group 4) then compared to the essential hypertension group (group 1) respectively. Mean stone size was 7.1 ± 8.8 mm in group 3 and 6.0 ± 5.5 mm in group 4, revealing a significantly larger renal stone size in BAH (p = 0.023) and APA (p = 0.028) groups than that in the essential hypertension group. The mean HU of the stone was 295.7 ± 226.5 in group 3 and 242.5 ± 178.8 in group 2. Although it did not show a significant difference, the PA group presented a tendency of higher HU values than the essential hypertension group.

**Table 3:** The size and Hounsfield unit of the stone comparing essential hypertension with bilateral adrenal hyperplasia and aldosterone-producing adenoma.

Characteristics	Subjects with essential hypertension N = 44	Subjects with primary aldosteronism N = 103			
		**Bilateral adrenal hyperplasia (BAH) N = 49**	**p value**	**Aldosterone-producing adenoma (APA) N = 54**	**p value**
Stone size (mm)	4.0 ± 3.3	7.1 ± 8.8	**0.023**	6.0 ± 5.5	**0.028**
Stone (HU)	223.8 ± 159.6	295.7 ± 226.5	0.083	242.5 ± 178.8	0.591

HU, Hounsfield unit. A p value < 0.05 is regarded as statistically significant (shown in bold).

## Discussion

Hyperaldosteronism is associated with hypercalciuria and hypocitraturia that are predisposed to nephrolithiasis (29). The biological effect of aldosterone includes promoting sodium and water retention and decreasing plasma potassium concentration (30). Owing to calcium being coupled to sodium uptake, body volume expansion will decrease sodium absorption in proximal convoluted tubules then influence calcium uptake (31). Although calcium will be reabsorbed in distal convoluted tubules, exorbitant excretion overpowers the absorptive capacity (32). Hyperaldosteronism results in volume expansion, inhibits proximal sodium and calcium reabsorption, and then contributes to hypercalciuria. Besides, the depletion of serum potassium also influences phosphate reabsorption. The decrease of phosphate stimulates calcitriol synthesis and then enhances intestinal calcium absorption or increased bone dissolution, resulting in hypercalciuria (33, 34). Due to low extracellular potassium concentration, potassium shifts out of the cells, and to maintain electrical neutrality, hydrogen shifts into the cells, raising blood pH and reducing intracellular pH. Intracellular acidosis increases the uptake and metabolism of citrate in the proximal tubules, leading to hypocitraturia (35, 36). Citrate binds with calcium to produce a soluble complex that can prohibit crystal nucleation and growth and lead to prevent nephrolithiasis (35). Taken together, the abovementioned electrolyte disorders such as hypercalciuria and hypocitraturia caused by PA could indirectly give rise to increased calcium nephrolithiasis.

Primary hyperparathyroidism (PHPT) is a common endocrine disorder that exorbitantly secretes parathyroid hormone (PTH) from the parathyroid glands. It is generally discovered when asymptomatic, but the disease always has the latency to become symptomatic, leading to kidney stones, cortical bone loss, and fractures (37). Elevated PTH increases urinary and plasma calcium levels by means of its effects on bone, kidney, and intestines. In the bones, it promotes osteoclastic bone resorption, leading to elevated plasma calcium (38), while in the kidneys, it facilitates renal tubular reabsorption of calcium; however, owing to increasing filtered load, the excretion of renal calcium typically overpowers the absorptive capacity (39). One multicenter cross-sectional cohort study discovered higher serum aldosterone concentration was associated with higher serum PTH concentration (40). A positive and bidirectional physiological relationship between aldosterone and PTH was also noted (41). Another study hypothesized that chronic exposure to aldosterone excites PTH as the mineralocorticoid receptor is expressed in the parathyroid, bringing about urinary calcium loss. Furthermore, PTH strengthens the aldosterone response to Angiotensin II, and PTH receptors are expressed in aldosterone-producing cells (42). It is important to realize that the homeostasis mechanisms of endocrine systems are complicated, with each single hormone possibly not being adequate to identify biological and clinical outcomes alone. The physiology of nephrolithiasis in patients with PHPT has not yet been elucidated, and the main mechanism still requires future investigation.

The first case report described the patient in whom bilateral renal calculi were present for several years prior to diagnosis of PA (28). After diagnosis, hypertension and persistent hypokalemia were corrected by spironolactone therapy, and following that, two-stage percutaneous nephrolithotomy (PCNL) was performed with no recurrent renal stone being noted during 12 years of follow-up. Shey et al. (29) discussed a patient with PA and recurrent renal nephrolithiasis where hypertension and hypokalemia were not well-controlled even with several medications, so due to medical treatment failure, the patient accepted adrenalectomy; finally, blood pressure returned to normal with medication and he remained stone-free for 10 years. Tantisattamo and Francis (43) also presented a previously normotensive, normokalemic, normocalcemic young man with urolithiasis in initial CT imaging but with no adrenal abnormalities. Three years later, he presented with severe hypertension and hypokalemia, and imaging showed an adrenal adenoma and recurrent nephrolithiasis found simultaneously, with hypertension being well-controlled after laparoscopic adrenalectomy. Chung et al. (44) conducted a nationwide cohort study and found that patients with PA were associated with a higher risk of urinary bladder stones than patients without PA (odds ratio, 1.68; 95% confidence interval, 1.20–2.34). The possible biological evidence was that aldosterone increased hypercalciuria and hypocitraturia. Also, some evidence suggests that aldosterone could influence calcium-activated potassium (BK) channels, which are indispensable in adjusting the function of bladder smooth muscle. Despite PA being considered as a risk factor for patients with nephrolithiasis, data regarding the risk of developing kidney stones in PA are still lacking, so further cohort study is needed.

A recent review of epidemiological data revealed that prevalence rates for kidney stones from seven countries were 1.7% to 14.8% (45). Additionally, prevalence rates appear to be rising. In the United States, there is developing evidence for an increasing prevalence rate of kidney stones from 3.2% in the period 1976–1980 to 8.8% in 2007–2010 (46). In our study, the incidence rate for kidney stones in patients with PA was near 24%, which was higher than that of a recent review. Also, there is significant variation in rates based on gender, age, geography, genetics, and numerous systemic diseases. However, hypertension being related to the incidence of nephrolithiasis is still controversial. Madore et al. (47) published a prospective cohort study and investigated the association between hypertension and nephrolithiasis. With 8 years of follow-up duration, a history of KSD was associated with a greater risk of developing hypertension (odds ratio, 1.29; 95% confidence interval, 1.12–1.41); however, hypertensive patients did not show a higher incidence of new stone formation (odds ratio, 0.99; 95% confidence interval, 0.82–1.21). Accordingly, our study used essential hypertension as the control group to compare with the PA cohort.

Kidney stone management depends on the symptom, the size, and the location. Whether asymptomatic kidney stone should be treated is still under debate. One review concerning asymptomatic calyceal stones revealed that average spontaneous stone passage rate and average requirement for surgical intervention were 18% and 20%, respectively. Also, 62% of patients remained asymptomatic on surveillance over a mean time of 4 years (48). Another systemic review included an asymptomatic kidney stone article over 25 years and indicated that stone size was not a reliable predictor of symptoms, but the need for intervention was linked to stone size. Stones >5 mm have 36% greater risk of intervention compared with stones of <5 mm (49). In the present study, the mean stone size in PA patients was 6.5 mm, which was greater than 5 mm; hence, the possibility of the need for stone intervention was higher than that in essential hypertension patients.

Several studies have indicated that kidney stone density can predict success in treatment. Stones with a mean density of >1,000 HU are less likely to be disintegrated by extracorporeal shock wave lithotripsy (ESWL) because the higher the stone density, the stronger the shock wave energy and the more the frequency needed to achieve fragmentation (50, 51). A threshold of 750 or 970 HU has also been reported (52, 53). Besides, Gücük et al. (54) investigated the effects of certain parameters including HU on the outcome of 179 PCNL patients and concluded that the HU value was an independent factor that affected the success of PCNL. Specifically, an HU value <677.5 reduced the success of PCNL by 2.65-fold. Although lower HU values are easily disintegrated by laser, such stone fragments are hard to be harvested completely; moreover, stones with lower density are more difficult to identify clearly under fluoroscopy. So, all the above reasons indeed decrease the stone-free rate. Besides, Gok et al. (55) also studied the effect of the HU value with a cutoff of 1,000 on the outcome of PCNL. In this study, there was no significant difference between low HU and high HU groups in terms of PCNL success rate, but duration of surgery and fluoroscopy was significantly higher in the high HU group because more time was required to disintegrate the high-density stones.

There are several limitations in our study. Firstly, it is a retrospective study with a small sample size, so further large and prospective studies are warranted to prove the validity of our findings. Secondly, environmental, diet habit, or genetic factors might influence stone formation, which were not evaluated. Confounding bias might be noted. Thirdly, this is a retrospective study of hypertension and PA patients, so we did not collect complete chemical analysis in the urine because we usually do not need to check urine analysis for hypertension and PA patients. Therefore, some potential association between urine and blood chemical variation cannot be confirmed. Fourthly, although renal stone presence was confirmed by abdomen CT, biochemical and radiological correlations were not evaluated. Finally, the influence of medication such as potassium or calcium supplements and diuretics was not assessed. Despite the aforementioned limitations, to the best of our knowledge, this is the first pioneer study to analyze the relationship between renal stones and PA. It could provide valuable results for further studies.

## Conclusion

The study demonstrated that PA patients present a higher incidence rate of renal stones compared with that in the general population. Besides, PA patients present larger kidney stone size than that in essential hypertension patients, including subgroups of BAH and APA. It also revealed that stone density of PA patients had higher HU values than that in essential hypertension patients, although not reaching significance. Because renal stones of PA patients present a larger size and a higher density, this might increase the difficulty of surgery. Further comprehensive research concerning the association between PA and renal stones is warranted.

## Data Availability Statement

The data during the current study are not publicly available due to privacy protection issues but are available from the corresponding author on reasonable request. Requests to access the datasets should be directed to H-YL, ashum1009@hotmail.com.

## Ethics Statement

The studies involving human participants were reviewed and approved by KMUHIRB-E(II)-20180184 and NTUH-REC No. 200611031R. The patients/participants provided their written informed consent to participate in this study. Written informed consent was obtained from the individual(s) for the publication of any potentially identifiable images or data included in this article.

## Author Contributions

C-KC and H-YL conceived the project. C-CC and V-CW collected the data. J-HG analyzed the results. C-KC and H-YL edited the article. All authors contributed to the article and approved the submitted version.

## Conflict of Interest

The authors declare that the research was conducted in the absence of any commercial or financial relationships that could be construed as a potential conflict of interest.

## Publisher’s Note

All claims expressed in this article are solely those of the authors and do not necessarily represent those of their affiliated organizations, or those of the publisher, the editors and the reviewers. Any product that may be evaluated in this article, or claim that may be made by its manufacturer, is not guaranteed or endorsed by the publisher.
